# Occurrence of metopic suture in modern and archaeological Croatian population

**DOI:** 10.3325/cmj.2024.65.174

**Published:** 2024-06

**Authors:** Tina Bareša, Ivan Jerković, Željana Bašić, Ana Curić, Goran Dujić, Krešimir Dolić, Šimun Anđelinović, Dragan Primorac, Ivana Kružić

**Affiliations:** 1 University Department of Forensic Sciences, University of Split, Split, Croatia; 2 University Hospital Center Split, Split, Croatia; 3 Museum of Croatian Archaeological Monuments, Split, Croatia; 4 St. Catherine Specialty Hospital, Zagreb, Croatia; 5 Faculty of Dental Medicine and Health, J.J. Strosssmayer University of Osijek, Osijek, Croatia; 6 School of Medicine, J.J. Strossmayer University of Osijek, Osijek, Croatia; 7 The Henry C. Lee College of Criminal Justice and Forensic Sciences, University of New Haven, West Haven, CT, USA; 8 School of Medicine, University of Split, Split, Croatia; 9 Eberly College of Science, The Pennsylvania State University, University Park, State College, PA, USA; 10 School of Medicine, University of Rijeka, Rijeka, Croatia; 11 Medical School REGIOMED, Coburg, Germany; 12 School of Medicine, University of Mostar, Mostar, Bosnia and Herzegovina

## Abstract

**Aim:**

To determine the incidence of metopism in the modern and archaeological Croatian population.

**Methods:**

A total of 800 specimens (454 modern multi-slice computed tomography [MSCT] scans and 346 dry archaeological skulls) were visually examined for metopic suture presence. The metopic suture was deemed complete when aligned nasion to bregma.

**Results:**

In the overall sample, the metopic suture was observed in 36 of 800 subjects (4.5%): 19 of 424 (4.5%) men and 17 of 370 (4.6%) women. A significant difference was not observed between modern and archaeological samples (χ^2^ = 3.219, *P* = 0.359) or between the sexes (χ^2^ = 0.006, *P* = 0.939). The frequency of metopism varied from 3.5% in the modern population to 7.04% in the samples from the Roman period.

**Conclusion:**

There are no visible secular changes on metopic suture in the Croatian population through time. Some variations can be the result of differences in sample size in different time periods.

The metopic suture is an additional suture that extends from the nasion to the bregma, separating the frontal bone into two halves (1). Metopic suture usually fuses in early childhood, but when visible after normal obliteration, it is called metopism and is considered a normal variation (2).

The main function of the metopic suture is to enable the movement of the frontal bone halves (3). Additionally, in comparison with other cranial sutures, the metopic suture serves less as a growth region (4). An important role in skull morphology (skulls with metopism tend to be wider) was reported in several studies (5-7), while others showed no connections between skull width and persistent metopic suture (8).

The obliteration of the metopic suture starts in the frontal tuber and extends to the nasion and bregma. The metopic suture closes from two ossification centers, but authors disagree as to the age when the closure starts. According to Vu et al (9), it starts at three months and is completed in nine months. Other authors suggested that fusion completes between ages two (10-12) and eight (13). The metopic suture can be complete (from the nasion to bregma) or incomplete (only the nasal part, only the parietal part, or both nasal and parietal parts but not connected) (14).

Premature fusion of the metopic suture can cause a rare form of cranial synostosis called metopic synostosis. This type of synostosis occurs in 5%-15% of all cranial synostosis patients who require surgical interventions (15-18). Additionally, the persistence of the metopic suture is associated with the developmental difficulties of the frontal sinuses (19,20), but Bilgin et al reported no association between the two (21). The connection between the persistent metopic suture and the closure time of the sagittal suture was shown by Nikolova et al (22). This study suggested that the closure time of sagittal suture was significantly delayed in metopic skulls, which makes the age estimation method less reliable (22).

From an anthropological standpoint, it is important that the frequency of metopism varies across populations. The frequency of the metopic suture varies from 0% to 15% depending on the population affinity and the sample size (2,8,23-32) (https://www.google.com/maps/d/embed?mid=1JUXqdyCpe21kbrn-16xJugGWJyiPxgk&ehbc=2E312F).

Although several studies have been conducted on European populations (Italian, Greek, Turkish, Romanian, English) (8,23,26,33,34), the frequency of the metopic suture in the Croatian population has not been assessed, as only one small study on the Roman period population was performed (35). The current study aimed to estimate the frequency of the metopic suture in the Croatian modern and archaeological population.

## Materials and methods

The archaeological sample consisted of 915 adult skulls from the Osteological Collection of the University Department of Forensic Sciences, University of Split. The sample was obtained from sites dating from the Roman period to the new age (Table 1). Preserved frontal bones were found in 345 cases (37.70%). The final sample consisted of 190 men (median age 40, range 18-69), 146 women (median age 30, range 18-60), and nine persons of unknown sex.

**Table 1:** The composition and demographic structure of the archaeological sample

Archaeological site	Period	Total adults	M	F	Unknown sex	Frontal bone	M	F	Unknown sex
Solin Smiljanovac (36)	Roman	605	257	226	122	142	66	68	8
Ostrovica greblje (37)	Medieval	46	30	16	0	36	20	16	0
Šopot Benkovac (38)	Medieval	19	15	4	0	15	12	3	0
Bijaći Stombrate (39)	Medieval	27	13	14	0	7	5	2	0
Svećurje Žestinj (40)	Medieval	17	11	6	0	10	8	2	0
Kučiće greblje (41)	Medieval	27	18	9	0	10	6	4	0
Kamenmost – Kaldrma (42)	Medieval	21	15	6	0	13	8	5	0
Gornji Koljani – Crkvina (43)	Medieval	30	17	13	0	8	6	2	0
Rižinice (44)	Medieval	5	4	1	0	1	1	0	0
Kaštel Stari Radun (45)	Medieval	97	51	45	1	84	43	39	1
Otok Vuletina rupa – Grebčine (46)	New age	21	16	5	0	20	15	5	0

The modern sample consisted of multi-slice computed tomography (MSCT) scans of the modern Croatian population. To capture within-population variations, the study included MSCT scans from Zagreb, the capital city located in the continental part of Croatia, and Split, the biggest city in the coastal region. A total of 454 MSCT scans (270 from the University Hospital Split and 184 from University Hospital Zagreb) were analyzed in OsiriX MD 12.0 (Pixmeo, Geneva, Switzerland) imaging software with the 3D volume rendering technique. The sample consisted of 229 women (median age 62; range 19-91) and 225 men (median age 62; range 20-91). Only adults from the Croatian population were included in the study. Persons with head trauma and obvious asymmetries were not included.

The persisting metopic suture in both archaeological and modern samples was deemed complete if the suture was aligned nasion to bregma. The subjects with incomplete metopic suture were not included, since a pilot study (data not shown) conducted on dry skulls and MSCT scans showed no agreement between visibility in different modalities (partial metopic suture was present in one case and was not visible on the MSCT scan).

### Statistical analysis

Counts and percentages of subjects with the metopic suture were calculated according to the results observed on dry bones and MSCT scans for the overall sample, each time period, and sex. Differences between the time periods and sexes were assessed with a χ^2^ test. The level of statistical significance was set at *P* ≤ 0.05. Statistical analysis was conducted with SPSS, version 22 (IBM Corp; Armonk, NY, USA).

## Results

The metopic suture was observed in 36 of 800 (4.5%) subjects: 19 of 424 (4.5%) men and 17 of 370 (4.6%) women. There was no significant difference between modern and archeological samples (χ^2^ = 3.219, *P* = 0.359) or between the sexes (χ^2^ = 0.006, *P* = 0.939). The frequency of metopism in different periods varied from 3.5% to 7.04%. The lowest frequency was observed in the modern population and the highest in the Roman period (*P* = 0.073; Table 2). In the archaeological sample, a complete metopic suture was visible in 20 subjects (12 men and 8 women; *P* > 0.05). In the modern Croatian sample, the persistent metopic suture was noted in 16 of 454 (3.5%) cases: 9 of 229 women (3.9%) and 7 of 225 men (3.1%) (χ^2^ = 0.224, *P* = 0.636). The distribution of the metopic suture by time period and sex showed no significant difference (Table 3).

**Table 2:** The occurrence of complete metopic suture (MS) in the archaeological sample

Period	Complete MS total (%)	Men (%)	Women (%)	χ2	P
Roman period	10/142 (7.04)	5/66 (7.57)	5/68 (7.35)	0.002	0.961
Medieval period	9/184 (4.9)	6/118 (5.08)	3/73 (4.11)	0.096	0.757
New age	1/20 (5)	1/15 (6.66)	0/5	n/a	n/a
Modern	16/454 (3.5)	7/225 (3.11)	9/229 (3.93)	0.224	0.636

**Table 3:** Comparison of the metopic suture frequencies according to the time period

	χ2	P
Roman – medieval	0.676	0.411
Roman – new age	0.116	0.734
Roman – modern	3.208	0.073
Medieval – new age	0.001	0.983
Medieval – modern	0.649	0.561
New age – modern	0.121	0.728

## Discussion

In this study, the metopic suture was observed in 36 of 800 subjects (4.5%). The incidence of metopism varied across different time periods, from 3.5% in the modern population sample to 7.04% in the sample from the Roman period. Although these two populations were most distant in time, there was no significant difference between them. To the best of our knowledge, studies that investigate secular changes in metopism occurrence in one population are rare. Only two were conducted on different populations: in today’s Turkey and Italy (8,23).

The Turkish study, conducted on the Anatolian population chronologically from the Neolithic to the 20th century, observed a higher incidence of metopism (between 3.33% and 14.3%), with the highest frequencies in the Hellenistic-Roman (14.3%) and 20th-century (11.6%) populations (8). The study on Italian-territory populations from the Roman and modern period also showed a higher frequency for the older (11.8%) than for the modern time period (5.8%-9.6%) (23).

The present study showed a slightly higher frequency in men in all studied time periods, except the modern, but no significant differences between the sexes were noted. In Anatolian populations, the frequency of metopism was higher in women when the total population was observed. When the population was divided according to time periods and settings, these frequencies varied, with no significant difference between men and women (8). The frequencies recorded in Roman and late-Roman samples from Daçta Burgaz (47) and Spradon (25) (both in Turkey) were 12.5% (11.1% women, 14.2% men) and 8.69% (13.2% women, 3.2% men), respectively. The present study showed similar results as the study on the Italian population from the same period (11.8%) (23), and although Italian and Croatian populations are geographically closer, the results from the Italian study are more similar to the results from Turkey. The results of Daçta Burgaz (47) and Spradon (25) populations are similar to those reported for the Anatolian population (8) from the same time period, while frequencies from the present study are lower. This result is expected considering the geographical location of the sites.

When compared with a 16-19th century Romanian sample (2.98%) (26), our new-age sample showed a higher frequency of the metopic suture (5%). The difference could be attributed to the geographical distance and secular changes since the Romanian sample has a longer time span.

Modern population studies conducted on Italian, Greek, and Lebanese populations reported various results (2,23,33); all except the Lebanese showed higher frequencies of metopism than the present study (3.5%). Studies conducted on different modern Asian populations showed results varying from 2.8% in the Thai sample (30) to 10.18% in the Chinese sample (32). The frequencies reported for different Italian geographical regions from the 19th century ranged from 5.5% to 9.6% (23); a similar result was shown on Greek donated bodies (7.5%) (33).

The modern Lebanese population (2), scored using radiographs, had the lowest frequency (0.82%; 8 of 968 persons had metopism). The low number of observations could be connected to the problems with the visibility of additional sutures on radiographs and the quality of scans (2).

The current study indicates that secular changes in different time periods do not remarkably affect the incidence of metopic suture. The variation in frequencies is the result of population differences and the power of statistical tests, considering the low frequency of metopism in general. However, despite the results not being completely conclusive, the present study is one with the greatest sample size in regard to the population size (see the map: https://www.google.com/maps/d/embed?mid=1JUXqdyCpe21kbrn-16xJugGWJyiPxgk&ehbc=2E312F). The study also considered the representatives of the major regions of the analyzed area. A study limitation is the structure of archaeological samples, all of which, except the Roman period sample, were collected from small cemeteries, so kinship cannot be excluded. However, this sample could well reflect metopism in ancient populations analyzed, as the samples were collected from a large skeletal collection containing 4000 specimens. Further studies should focus on temporal and spatial gaps in the existing literature to better understand the importance of metopism and its specificities in the clinical and forensic context.
